# The Movement-Image Compatibility Effect: Embodiment Theory Interpretations of Motor Resonance With Digitized Photographs, Drawings, and Paintings

**DOI:** 10.3389/fpsyg.2018.00991

**Published:** 2018-06-19

**Authors:** Mark-Oliver Casper, John A. Nyakatura, Anja Pawel, Christina B. Reimer, Torsten Schubert, Marion Lauschke

**Affiliations:** ^1^Excellence Cluster “Image Knowledge Gestaltung. An Interdisciplinary Laboratory”, Humboldt-Universität zu Berlin, Berlin, Germany; ^2^Working Group “Morphology and the History of Forms”, Department of Biology, Humboldt-Universität zu Berlin, Berlin, Germany; ^3^Working Group “General and Experimental Psychology”, Department of Psychology, Martin-Luther-University Halle-Wittenberg, Halle, Germany

**Keywords:** embodied cognition, situated cognition, image perception, compatibility effects, body schema, motor resonance

## Abstract

To evoke the impression of movement in the “immobile” image is one of the central motivations of the visual art, and the activating effect of images has been discussed in art psychology already some 100 years ago. However, this topic has up to now been largely neglected by the researchers in cognitive psychology and neuroscience. This study investigates – from an interdisciplinary perspective – the formation of lateralized instances of motion when an observer perceives movement in an image. A first step was to identify images that evoke a perception of movement in a certain direction and to give this a rating. Reaction times leading to the engagement of a joystick following the presentation of images are used to evidence the postulated movement occasioned by the perception of movement in an image. Where the required direction of joystick moves matched the expected perception of movement direction in the image, significantly shorter reaction times were recorded. The experiment was able to prove a “movement-image compatibility effect” in observers of images. Based on this, the paper revisits and brings up to date the theses on motor sensory response to images which were developed in art psychology at the beginning of the 20th century. It furthermore contributes an embodiment theory interpretation to the prevalent representational explanation of compatibility effects.

## Introduction

In 1922 Paul Klee asked, “What does movement in the work actually mean? Our art is not capable of movement, is it? We are not fabricators of automatons, after all! No. Our works will by their very nature be confined to one place and stay there. And yet they are all about movement” (Transl. by the author, Klee and Glaesemer, 1979, p. 94). To evoke the impression of movement in the “immobile” image is one of the central motivations of the visual arts; at the same time, it is a seemingly impossible task. In the interaction between artist and image, but also in the processes of perception between image and observer, the body and its potential for movement seems to play a vital role. The present study has been developed by an interdisciplinary group and is dedicated to the examination of this subject.

In art psychology, research of the processes that are involved in the perception of movement in the image is a central concern (e.g., Arnheim, 1966). It is one to which cognitive psychology and neuroscience have generally paid little heed, despite the fact that insights into the activating effect of images have for some time been available (Lauschke, 2014, 2016, 2017a,b). In art history, efforts to understand the activating effect of images led to the formation of a “picture act” theory, which understands images as agents (Bredekamp, 2010). In recent years eye-tracking studies have been used in connection with the observation of works of art and for the first time art historical research was combined with psychology and cognitive science (for example, Rosenberg and Klein, 2015). Our project aims to expand empirical image studies by providing more information on the role of the entire body and its motion response to image perception.

Our study is a contribution to empirical investigations related to the issue of “embodied cognition.” Many experiments, using diverse methods, have been carried out in the pursuit of various phenomena in the same context. Such phenomena include: linguistic competence (Barsalou et al., 2003; Pulvermüller et al., 2004; Louwerse, 2008; Rueschemeyer et al., 2010; Glenberg and Gallese, 2012; Pulvermüller, 2013), the perception of space (Holmes and Spence, 2004; Cardinali et al., 2009a), multi-modal integration (Sirigu et al., 1991; Maravita et al., 2003), the use of instruments (Cardinali et al., 2009b) and the perception of objects (Tucker and Ellis, 1998). Experimental work has, however, been cautious in tackling the subject of image perception. To date, there are few publications in this field (Freedberg and Gallese, 2007; Proverbio et al., 2009; Umiltà et al., 2012; Sbriscia-Fioretti et al., 2013; Ferretti, 2016).

This study does not limit itself to a “moderate embodied cognition approach” (Goldman, 2012). It proceeds on the premise of a “full-blown” embodiment theory, which argues that even the *non-neural* parts of a body contribute to the constitution of *cognitive* conditions and processes. Such a premise gives rise to certain methods. If cognition in general and thus also perception – or the perception of movement in the image – are to be understood as integral to the physical equipment of a cognitive organism, then empirical embodiment theory studies must take the entire corporeality of a cognitive organism into account, in order for questions on specific cognitive abilities to be answered. Neuroscientific methods (such as EEG or fMRT) require the subject to be immobile. This inhibits the very motor resonance between the perception and performance of movement which is at the heart of our study. For this reason, we decided to use reaction time measures in our study.

A further consequence of the above-mentioned embodiment theory premise is that interdisciplinarity becomes an essential factor in addressing questions of cognitive science. For example, in cognitive neuroscience, the body *must* play a subordinate role since cognitive neuroscience focus on neural structures and activities. Yet in studies that explicitly examine the body, such as biology or human anatomy, the requirement is mostly absent that *cognitive* phenomena be explained through research findings (Shapiro, 2004). A challenge therefore arises: results from various disciplines must be combined in such a way as to give credence to cognitive science theories without restricting evidence to that found in neural networks and activities.

In cognitive psychology, “compatibility effect” is a term describing, among other things, the phenomenon whereby the location of a stimulus co-determines the type of response to it (Umiltà and Nicoletti, 1990). According to Kornblum et al. (1990), this can be perceived as resulting from overlapping spatial dimensions of the physical world and of movement dimensions of the behaving body (Dimensional Overlap Model, DOM). In experiments it has been found that in situations in which the location of a stimulus and the direction of required action are congruent, reaction times are shorter than in the opposite case. The phenomenon has come to be known as the “Simon effect” (Simon, 1990) and it is conceptualized by Kornblum et al. (1990) proposing the DOM. A similar effect was observed by Glenberg and Kaschak (2002) in respect of motor reactions to spoken stimuli (“action-sentence compatibility effect”).

In the theoretical contexts of art psychology and esthetics, theories were developed between 1880 and 1930 which may be thought of as preparing the way for today’s research into compatibility effects. It should be stressed, however, that those earlier theories were formulated from a different perspective than current research into “compatibility effects.” Whilst original concerns were directed to understand the esthetic experience of images, cognitive psychology seeks to investigate effects on behavior. In the late 19th century, Wölfflin established a link between art history and psychology. He brought the human body into focus in its significance for the perception of art. Wölfflin (1886) formulated what was for his times a daring hypothesis, assuming that visual stimuli are directly conducted to the motor apparatus. Only much later would this be adopted by cognitive psychology. One source of his ideas came from the esthetics of empathy (Einfühlungstheorie). According to Vischer (1873), mental agitation in perception was apparent in physical movement. Vischer explained “empathetic motor response” as a factor of the close interdependence of the visual and tactile senses. Lipps (1923, p. 144) maintained that forms prompted impulses toward movement in the observer. He also made clear that the same vitality that observers notice in their own or other human bodies could be ascribed to figures in images. From our current perspective, the cited work of Wölfflin, Vischer, and Lipps not only resembles the assumptions of embodiment theory but may also be understood as a collection of pioneering theories in the field of “compatibility effects.”

Laboratory findings by Münsterberg (1889), a pioneer in applied and experimental psychology, confirm the presumption of a direct connection between sensory and motor systems, as well as of the existence of involuntary bodily responses to perceived stimuli. The art psychologist and philosopher Müller-Freienfels drew on Münsterberg’s findings (1922). He coined the term “motor resonance” to capture instances of movement in response to the perception of movement in the image on the part of a beholder. His criticism, now some 100 years old, of “psychological intellectualism that seeks to explain everything by representation” (Transl. by the author, Müller-Freienfels, 1922, p. 118) has in the last three decades become a subject of debate once more, following a turn toward the body in philosophy and in the cognitive, art and cultural sciences. The term “motor resonance” is today applied in neuroscience (Uithol et al., 2011) and in cognitive psychology (Schütz-Bosbach and Kuehn, 2014) – albeit without reference to Müller-Freienfels. Müller-Freienfels’ “motor resonance” and Münsterberg’s efforts have opened a horizon in theory that allows a very close connection between perception and body, between sensory and motor systems, and between stimulus and response (such as occurs in connection with compatibility effects) to be substantiated.

In the 1990s the working group of Odmar Neumann in Bielefeld attempted to prove the potential for direct motor activation through visual stimuli independently of conscious visual perception (“response priming”) and to confirm Münsterberg’s hypotheses using modern experimental psychology and electro-physiology (Ansorge et al., 1998; Neumann et al., 1998; Klotz and Neumann, 1999; Ansorge and Leder, 2011; see also Schubert et al., 2013). The common coding theory developed by Prinz and others (Prinz, 1990) and Hommel’s event coding theory (Hommel et al., 2001) are based on the assumption that perception and action are directly linked via a common code. This theory suggests that motor functions are occasioned by the mental representation of the effects of anticipated action. Both theories refer to stimulus-response compatibility and explain this by proposing that stimulus information releases “lateralized readiness potential” and even gives rise to subliminal movements associated with the presented stimulus (Eimer et al., 1995; Hommel, 1997).

Freedberg and Gallese (2007) were the first to employ neuroscientific methods to investigate bodily reactions to images. They posited that the motive effect of images can be attributed to bodily resonance and results from the activation of embodied mechanisms, i.e., neural-based automatic simulation of actions, emotions and bodily sensations. Proverbio et al. (2009) showed that photographs portraying intensely dynamic actions possessed a strong cortical activation potential, compared with less dynamic images. Ferretti (2016) argues that “action properties” may be attributed even to objects portrayed within images. In 2012 a research group examined whether looking at abstract art could release motor resonance (Umiltà et al., 2012). Using EEG, measurements were taken of the changes in brain waves of beholders of works by the Italian artist Lucio Fontana. Significant suppression of μ-rhythms was observed in beholders of Fontana’s work, with its slashed canvases. No suppression of μ-rhythms was observed in control experiments using graphically modified versions of Fontana’s work. The authors of the study drew the conclusion that looking at the slashed canvas prompted the same activation of the motor cortex as would be triggered in a person observing, or performing the action of slashing. However, it should be noted that the reactions to Fontana’s images cannot be generalized in respect of abstract art; the peculiarity of his images consisting in the fact that the slashes are “real” rather than “abstract.”

**FIGURE 1:**
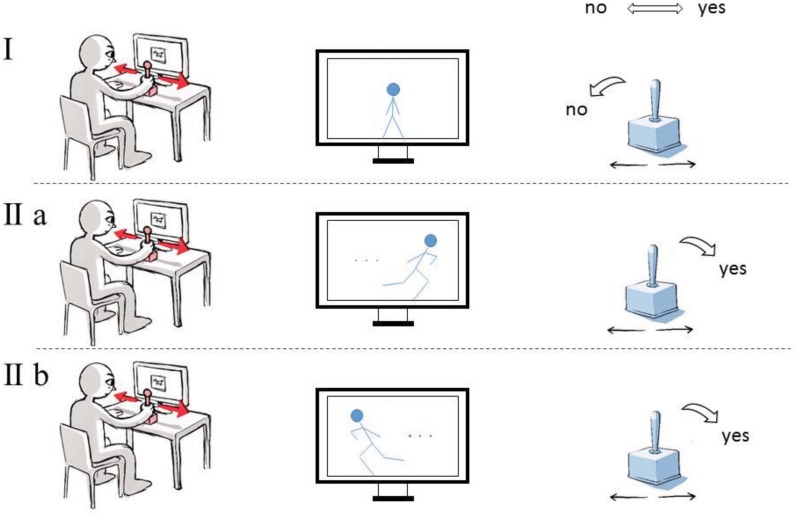
The experiment on the perception of movement in images. Participants were asked to indicate by moving a joystick whether they perceived a suggestion of movement in the image **(IIa,b)** or not **(I)**. Shorter reaction times were anticipated if the required joystick move was compatible with the perceived direction of movement **(IIa)** than in the contrary scenario **(IIb)**.

The present study is linked to the investigation on image perception and refers to the assumptions that have emerged over the course of some 100 years now accepting the existence of motor resonance between images and their beholders. The aforementioned premise of the study, based on embodiment theory, will take this pioneering work into account. We pursue a “full-blown” embodied cognition approach that is, on the one hand, not only historically contextualized but also theoretically supported by art historians (Bredekamp, 2010) as well as philosophers (Gallagher, 2005; Krois, 2011). On the other hand, it is embedded in empirical research that uses different methods and experimental designs than commonly applied for studying similar issues. By conducting the study in this way, we hope to shed new light on the issue of image perception. The experiment set-up borrows from a study conducted by Glenberg and Kaschak (2002) in order to develop the line of empirical and embodied cognition research further. For that purpose, we aimed to measure an image-compatibility effect, when participants observe digitized photographs, drawings, and paintings that show movement. In particular, we asked participants to judge whether or not an image involved a movement impression by moving a joystick to the left or to the right side. Subsequently, we divided the presented images in images with a left- or right-sided movement direction and analyzed the reaction times of participants depending on the compatibility relation between the yes or no-movement response and the direction of the movement in the presented image (i.e., left or right-sided movement direction). We expected decreased response times in compatible conditions (i.e., conditions in which the side of the yes-movement response coincided with the movement direction of the presented image) compared to incompatible conditions. In the first step of the investigation, we identified images that evoke a perception of movement. In the second step of the investigation, we measured the reaction times from the presentation of images to the engagement of a joystick (“joystick move”) (**Figure 1**).

## Materials and Methods

### Participants

All procedures performed in the experiment were in accordance with the standards of the ethics committee of the Humboldt-Universität zu Berlin and with the 1964 Helsinki declaration and its later amendments. The ethics committee of the Humboldt-Universität zu Berlin approved the study. Written informed consent was obtained from all participants. Separately, 25 people took part in the rating of selected images (14 women, 11 men) with an average age of 24.7 years (*SD* = 4 years; ages in the range of 18–36 years). The experiment itself involved 18 participants (8 women, 10 men) with an average age of 25.2 years (*SD* = 2.1 years, ages in the range of 21–29 years). All participants were recruited from the participant database of the Department of Psychology at Humboldt-Universität zu Berlin, were registered and received eight Euro per hour for compensation. Prior to the experiment, a standardized handedness test was used to ensure that all participants were right-handed (Oldfield, 1971). The participants passed the test with a mean laterality quotient of 71.72 ± 23.21.

### Image Selection

**FIGURE 2:**
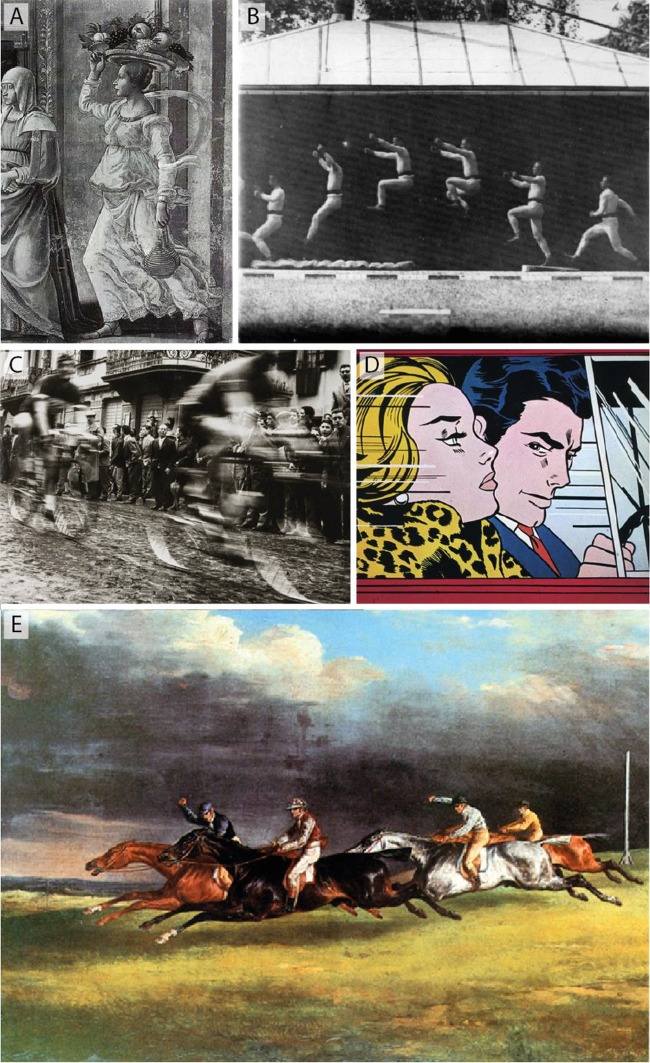
Examples for images with perceived movement. **(A)** “Bewegtes Beiwerk”, i.e., apparently moving clothes or hair. **(B)** Chronophotographic image sequence. **(C,D)** Motion blur. **(E)** “The Derby of Epsom” by Théodore Gericault.

Image selection was performed on the basis that an image either evoked the perception of movement that was specifically directed toward the left or right (edge of the image) or that it gave the impression of a resting position – in other words, these images did not suggest any direction of movement. Photographs, paintings and drawings from a variety of art periods were considered. Most images depicting movement contained characteristics of a posture and position of a moving figure (humans and/or animals), such as a rolling movement of the foot, shifting of weight or the impression of a movement about to be, or having been undertaken as well as the positioning (of the body) in the picture and the orientation of a figure and direction of gaze, including in relation to composition or perspective. The following indicators of movement were chosen: (i) “Fluid” clothing. The art historian Aby Warburg developed the term “bewegtes Beiwerk” hence the undulation of hair and clothes enhances the impression of an inner motion of a figure (see **Figure 2A** as example). (ii) Superimposition of single sequences of movement (such as in multiple exposure images). The superimposition of single sequences of movement plays a crucial role for evoking motion as it was already developed in chronophotographic studies in 19th century (see **Figure 2B** as example). (iii) Traces of movement such as fuzziness, blurring, lines (see **Figures 2C,D** as examples). An image might fulfill several of these criteria. In the example of the “Derby of Epsom” by Théodore Gericault (1821) some of these criteria can be seen, even if the running horses anatomically are not correct (**Figure 2E**).

The images were then evaluated by independent observers on a scale from -5 (perceived movement to the left) to +5 (perceived movement to the right), with the “0” reserved for images that did not suggest movement of any kind. 16 images were selected that had been evaluated at “0” by every observer and were used as control images (hereafter “static images”). Fifteen images received an average rating value of more than +2.8 or less than -2.8 and were subsequently included in the experiments. The results of the rating show that mostly images with figural motifs were able to evoke a distinct impression of movement toward a certain direction in the observer. Abstract motifs, even if demonstrating specific directions of movement or a tendency within the composition format through arrows or lines, were insufficient to produce the same impression. However, it should not be concluded that abstract images are in principal unable to evoke the impression of movement. It might well be that the specific questions of the rating made it hard for abstract images to be considered for the experiment.

### Apparatus and Stimuli

The 15 images with the highest average evaluation of perceived movement were each reproduced as horizontal mirror images. The data set thus comprised 30 images with perceived movement and was balanced in respect of the direction of perceived movement and intensity of perceived movement in a direction. Together with the 16 static images this made a final data set of 46 stimulus images. The experiment was programmed using “Presentation” (Version 17.0, Neurobehavioral Systems, Inc. 2016). All 46 images were produced in a similar size of approximately 20 × 20 cm and presented with a central viewing distance of 70 cm against a dark background (CIE: *x* = 0.313, *y* = 0.329; luminance = 12.46 cd/m^2^). The screen was a 24 inch LED-TFT monitor with a resolution of 1920 × 1080 pixels and a refresh rate of 100 Hz.

### Design and Procedure

The 30 movement images and 16 static images were presented in a random sequence, in two blocks (i.e., 92 images were presented to each participant). The participant’s gaze was directed with the help of a small cross in the center of the screen. With the eye fixated during 500 ms, each image was presented centrally in front of the participant for 150 ms. Following reaction to the image, an empty dark screen was shown for 5000 ms during the inter trial intervals. The participants trained six exercise trials before the experiment. This was followed by the first block and, after a short break, the second block. The participants were asked to use the joystick to indicate if they had perceived any movement in the image and to do so as fast and correct as possible. For half of the participants, a joystick movement to the left was chosen to indicate positive confirmation, i.e., movement present (group A) and a right movement indicated no movement present in the image. For the other half of the participants, the response assignment was reversed (group B). Reaction times were measured from the presentation of the image to the first measurable move of the joystick. The direction of the first measurable move of the joystick was also registered.

### Statistical Analysis

**FIGURE 3:**
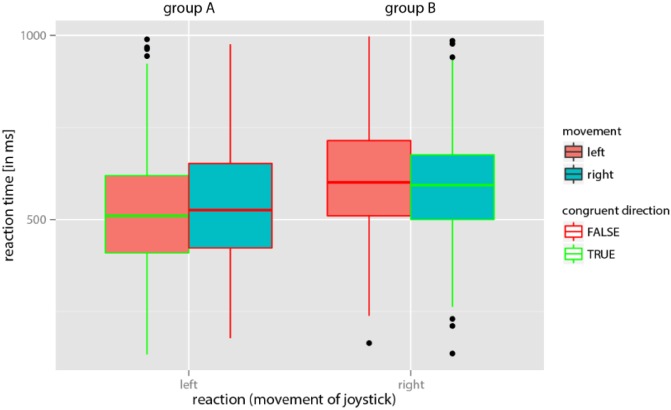
Reaction times in groups A and B for the perception of movement in the image as confirmed by the joystick move. Note that in group A participants were asked to indicate a perceived movement by moving the joystick to the left and in group B participants were asked to indicate perceived movement by moving the joystick to the right. In both groups, reaction times were on average shorter if the anticipated direction of perceived movement in the image was the same as that of the required joystick move (i.e., in the case of congruent directions).

A total of 1,656 reaction times (46 images × 2 blocks × 18 participants) were collected. A linear mixed model was calculated and depicted using the free statistics software “R” in “R Studio” (R statistics version 3.3.2; R Development Core Team (2018)) using several packages (*plyr*, *lme4*, *ggplot2*, *effects*, and *car)*. The model was used to check the data set for the postulated movement-image compatibility effect. In addition to the hypothesized effect of the compatibility of the expected perception of movement in the image and the reaction times of participants, we included the number of completed trials (for “repeated exercise” effects) as fixed effects in the model. Because joystick movements of the right handed participants generally were faster to the left independent from the perceived movement in the images (cf. **Figure 3**), we treated both groups (A and B) separately to study the hypothesized effect of movement-image compatibility. Joystick movements that did not correspond to expectations were classified as errors and removed from the analysis. However, it should be emphasized that an unexpected direction in joystick movements might be explained by a corresponding perception in the participant, and therefore potentially did not constitute an error. Participant No. 7 was excluded from the analysis due to extremely high variance in reaction times.

The following steps were carried out when analyzing the statistics. First, the “R” function “lmer” was used to establish a *linear mixed model*, which is suited to account for fixed and random effects. The model had the following formula (explained below):

$$\text{log}(RT)∼\text{poly}{\left(\text{Trial, 3}\right)}^{*}\text{Block}+{\text{Movement}}^{*}\text{Reaction+}(1+\text{Reaction+Movement|participant})+\left(1|\text{image}\right)$$

Since normal distribution was not given in the raw reaction times, normal distribution of data was subsequently achieved using logarithms of reaction times [the dependent variable *log(RT)*]. On the left side of the tilde (*∼*) the independent variables are coded. The term “poly(Trial,3)^∗^Block” in fixed effects is a third grade polynomial that approximates the development of reaction times over the duration of the experiment; i.e., the “repeated exercise” effect. “Movement^∗^Reaction” is the expected movement-image compatibility effect (i.e., a fixed effect, too). The model also takes into account random effects attributable to the individuality of the participants (the term *1 + Reaction + Movement| participant*) as well as the variation in strengths of perception of movement when looking at individual images (term *1| image*).

Besides normal distribution which was achieved by using logarithmized reaction times and to further verify the data structure preconditions (i.e., the assumptions) of the mixed model, a residual plot was produced (not shown). Residuals with values above 0.5 were removed (elimination of outliers). Similarly, steps were taken to ensure there was homoscedasticity of variance.

Finally, significance was checked using a *log-likelihood based Chi^2^ test* (χ^2^). This involved the exclusion of individual terms from the model; a comparison of the resulting model was made to establish which term had a significant effect. Furthermore, a confidence interval (CI) was produced.

## Results

Of the total number of 1,564 registered joystick movements (after the exclusion of participant No. 7) only 45 failed to comply with the expected direction of reaction based on the rating – which corresponds to an error rate of approximately 2.9% (on average 2.65 ± 3.44 errors out of 92 required reactions per participant). In 18 cases a perceived movement was confirmed by the joystick even though the rating had determined that the image in question did not evoke a perception of movement. In 27 cases, the joystick move indicated that there was no movement perceived in the image even though the stimulus was considered to be an image evoking the perception of movement. In only seven of these 27 cases (0.4% of all required reactions) the joystick was moved in a direction other than that determined by the rating.

**FIGURE 4:**
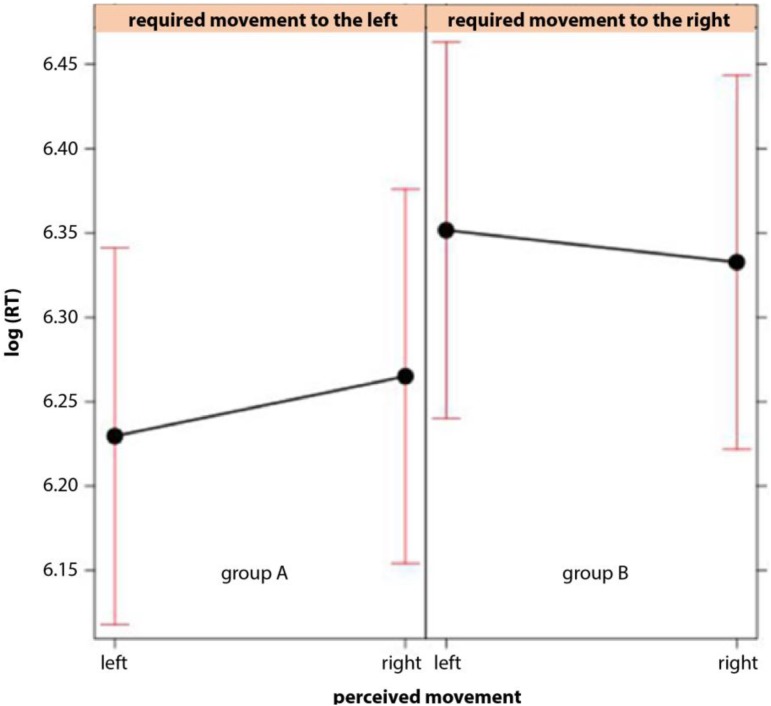
Effect plot of the interaction in the mixed model between the direction per expected perception of movement in the image as established in the rating and the required direction of the joystick move in confirmation plotted for groups A and B separately. Logarithmised reaction times [log(RT)] were used for the model. The model yielded a significant effect of interaction.

Consistent with the expectation, both groups (A and B) responded faster in the compatible condition (perceived movement in the image and required direction of joystick movement are congruent) than in the in-compatible condition (**Figure 3**). This observation was confirmed by the linear mixed model (**Figure 4**). Logarithms of reaction times were significantly smaller in the compatible condition (χ^2^ = 4.71; *p* = 0.03) than in the in-compatible condition (CI range from 2.5%: -0.0257 to 97.5%: -0.0015).

The duration of the experiment also had a significant influence on the result (χ^2^ = 17.70; *p* < 0.001). A repeated exercise effect was thus confirmed over the course of the experiment.

## Discussion

The aim of this study was to use the measurement of reaction times from presentation of an image to joystick move to prove an instance of physical movement caused by the perception of movement within an image. The primary finding of this experiment was the so-called “movement-image compatibility effect.” Analysis of the reaction times shows that participants require more time to give their answers where the perceived movement within the image is incompatible with the movement of the joystick required to give the answer (cf. *response priming*: Fehrer and Raab, 1962; Rosenbaum and Kornblum, 1982). The phenomenon involved here is also known as “motor resonance”. The following will discuss the results. The discussion will center on two possible interpretations of the results, both of which refer to the tradition of embodiment cognition research.

### Multi-Modal Representations and Priming

The stimulus-reaction pattern found in the experiment could be explained by focusing on the neurophysiological structure and its activity that is involved in the translation of stimulus into reaction. The claim is that perceptive input is neurally represented and processed by the activity of networks of neurons that are also partly responsible for preparing and controlling motor output (see DeCharms and Zador, 2000 on the term “neural representation”). A variation in reaction times to given stimuli is thus to be expected. The simultaneous representation, processing and preparation of both stimulus information and required motor reaction to the stimulus leads to interference in the cognitive systems of recipients, which in turn leads to longer reaction times. Attempts to explain compatibility effects in this way are often based on the assumption that both sensory stimuli and motor reactions are represented by *internal*, *multi-modal*, and *neural* representations; see “bodily formatted representations” (Goldman and de Vignemont, 2009; Goldman, 2012), “grounded cognition” (in Barsalou et al., 2003), or the “common coding theory” (Eimer et al., 1995). *Bodily formatted representations* (henceforth *B-formatted representations*) are neurally constituted, body-specific representations. They contain somatosensory, affective, interoceptive and motor-related information; they are *multi-modal* representations. Presumably, stimulus processing partly depends on such information. Hence, motor actions and sensory attributes might well be linked to each other via this kind of neural representation. For this to be true, it would mean that the neural components of the brain that generate body-specific representations also contribute to the execution of perception processes.

In the context of Goldman’s “moderate embodied cognition” approach and the idea of B-formatted representations, the neurophysiological parts one would expect to be involved in bringing about the movement-image compatibility effect are, on the one side, the visual cortex in the occipital lobe (Brodmann areas 17, 18, 19) since these neural areas are responsible for visual processing. However, because the proposal is that visual information might also be represented by the activity of motor-related neural networks, the primary motor, premotor, and supplementary motor cortex (Brodmann area 4, 6, and 44) (Pulvermüller, 2013) are very likely to contribute to the processing of visual stimuli and hence to play a vital role for constituting the movement-image compatibility effect, too. Since it is assumed that B-formatted representations are distributed over the *networks of mirror neurons*, they are brought about by similar neural structures as mirror effects are (Goldman, 2012). Mirror effects relate to phenomena whereby the mere observation of an action results in activation of those neural areas that would be required to carry out the observed action (Keysers et al., 2003; Avenanti et al., 2007; Keysers, 2009). Mirror effects are evoked by so-called mirror neurons (Rizzolatti et al., 1996; Keysers et al., 2010) and they are sometimes understood as a phenomenon of neural reprocessing or redeployment (Anderson, 2008, 2010; Goldman, 2012). The core networks of mirror neurons in humans are, according to Rizzolatti and Craighero (2004, p. 176) formed by “occipital, temporal, and parietal visual areas, […] the rostral part of the inferior parietal lobule and the lower part of the precentral gyrus plus the posterior part of the inferior frontal gyrus.”

Altogether, the concept of B-formatted representation links the sensory and motor systems in such a manner that experiments investigating the perception of movement in the image, such as those described here, evoke interference in the cognitive system of the recipient. This leads to prolonged reaction times, particularly in cases where a given stimulus activates the sensory-motor neuron system in such a way that the participant must first overcome an activation impulse in order to execute the target reaction.

### Alternative Interpretations: Movement-Image Compatibility as Body Schematic Response

For some 30 years, a debate has been developing on the topic of “situated cognition” involving various disciplines such as psychology, philosophy of mind, neuroscience and artificial intelligence studies. Current research of situated cognition has coined such terms as “extended mind,” “embedded cognition,” “enactivism” and “embodiment theory.” Some theories behind those terms have in common that they reject today’s most dominant cognitivist view that cognition is an input-output conversion that can be couched in representational terms (e.g., Hutto and Myin, 2013, 2017). A specific group of approaches among those critical theories emphasize the constitutive role of the body, or non-neural parts of the body for cognitive processes. They focus on a special understanding of the “body schema” in order to understand how cognitive processes like visual perception arise (Gallagher, 2001, 2005, 2012, 2015; Krois, 2011). The extended reaction times to given stimuli that were observed in the experiment might be explained by a “full-blown embodied cognition” approach that refers to the body schema of participants that is affected by the perception of movement in the image.

The term “body schema” was prominently introduced by the neurologist Head (Head and Holmes, 1911), who saw it as a model of physical posture. Nowadays, it is still used in sensorimotor cognition theories (for an overview of these, see for instance Bishop and Martin, 2014). It is important to stress that there exist several versions of the concept of the body schema (Cardinali et al., 2009a). In many studies, aspects of the body schema (such as body ownership or peripersonal space) are cited in the investigation of sensory and multi-sensory processes (Makin et al., 2008; Ehrsson, 2012). Other studies identify the “self” with the body schema (de Vignemont, 2007) and examine aspects of a person’s consciousness, among other things. The conception proposed in the following refers to the analysis of (multi-) sensory processes. It does not necessarily accept representationalist theories which in several sources are linked to the body schema (e.g., Makin et al., 2007). Krois (2011) develops this term and redefines it as a subpersonal, multi-sensory system that automatically controls posture and movement. It governs reflexes, physical progression, instrumental movements such as grasping, and expressive movements such as gestures (Gallagher, 2005); it therefore also governs the joystick moves that were required of participants in the experiment. The body schema is not identifiable as a single part of the body. It is holistic because it is the coordination of information won from diverse parts of the body and from diverse sensory processes. It coordinates visual, haptic and proprioceptive information such that, for instance, bodily balance can be maintained. In order to conduct the coordination of sensorimotor processes, the body schema must fulfill two functions. It must integrate retentional as well as protentional aspects of movement. A change of position just carried out (the so-called retentional aspect of an actual movement) influences which motor procedures can be subsequently performed (the protentional aspect of movement). The coordination of retentional and protentional aspects of motor procedures are made possible through the integration of diverse sensory information. Krois posited that images give rise to the perception of dynamic relationships as vital forces. Bredekamp (2010) investigated the interconnection of embodiment and the activating effect of images. The claim is that processes of the body schema such as the maintenance of balance, the understanding of spatial relations, and the implicit knowledge of one’s own ability to move are also at work when observing an image. In the case of perception of images that show movement, the visual information is treated by the body as motor information; so a motor disposition is built up in recipients on a subpersonal level that puts the observer in a state of readiness. This leads to extended reaction times in those cases where the required joystick move in the experiment does not conform to the direction of movement in the image.

## Conclusion

Our experiment on the perception of movement in images was able to show evidence for the motor resonance of images. The proven effect is called “movement-image compatibility effect.” The experiment also provided further confirmation that the effects and perception of images nonetheless remain complex and challenging areas. As a result of the rating conducted as part of the selection of images and before the experiment itself, it became apparent that mostly figural motifs had been identified as suggesting movement. In the rating, images with a more complex structure such as abstract motifs were insufficient to produce a marked impression of movement in the observers. This is also the case if they demonstrate specific directions of movement or at least tendencies within the composition format through arrows or lines. Future research should aim to explain these circumstances, which are far from being fully understood.

The discussion on a possible explanation of the movement-image compatibility effect and similar effects furthermore showed that various disciplines have put forward consistent hypotheses with arguments at diverse levels. It is not possible to come to a conclusion as to whether significantly longer reaction times in the experiment presented here could be caused by neural interference, by multi-sensory integration or by a concatenation of both. However, the term “body schema” allows us to include the entire biological equipment of a cognitive organism, and not just its neurophysiological structures, when investigating cognitive abilities such as perception or the perception of movement in the image. This does not mean that experimental research into cognition can rely solely on the testing of behavioral hypotheses. The brain plays an important role in the constitution of cognitive states and processes. Only through neuroscientific methods can the details of brain functions or areas of the brain be discovered and understood. The challenge is in integrating the results in a broader debate about what constitutes cognitive states and processes like visual perception of images.

## Author Contributions

AP, JAN, M-OC, and ML contributed conception and design of the study. AP, JN, M-OC, and ML organized the database. JAN performed the statistical analysis. TS reviewed the statistical analysis. CR and TS enabled the performance of the experiment. AP, JAN, M-OC, and ML wrote sections of the manuscript. CR and TS revisited and commented the manuscript. All authors contributed to manuscript revision, read and approved the submitted version.

## Conflict of Interest Statement

The authors declare that the research was conducted in the absence of any commercial or financial relationships that could be construed as a potential conflict of interest.
